# Assessing Collagen D-Band Periodicity with Atomic Force Microscopy

**DOI:** 10.3390/ma15041608

**Published:** 2022-02-21

**Authors:** Andreas Stylianou

**Affiliations:** Cancer Mechanobiology and Applied Biophysics Group, Basic and Translational Cancer Research Center, School of Sciences, European University Cyprus, Nicosia 2404, Cyprus; an.stylianou@euc.ac.cy; Tel.: +357-2271-3043

**Keywords:** collagen, Atomic Force Microscopy (AFM), D-band, D-periodicity, D-spacing, mechanical properties, biomaterials

## Abstract

The collagen superfamily includes more than fifty collagen and/or collagen-like proteins with fibril-forming collagen type I being the most abundant protein within the extracellular matrix. Collagen type I plays a crucial role in a variety of functions, it has been associated with many pathological conditions and it is widely used due to its unique properties. One unique nano-scale characteristic of natural occurring collagen type I fibers is the so-called D-band periodicity, which has been associated with collagen natural structure and properties, while it seems to play a crucial role in the interactions between cells and collagen and in various pathological conditions. An accurate characterization of the surface and structure of collagen fibers, including D-band periodicity, on collagen-based tissues and/or (nano-)biomaterials can be achieved by Atomic Force Microscopy (AFM). AFM is a scanning probe microscope and is among the few techniques that can assess D-band periodicity. This review covers issues related to collagen and collagen D-band periodicity and the use of AFM for studying them. Through a systematic search in databases (PubMed and Scopus) relevant articles were identified. The study of these articles demonstrated that AFM can offer novel information concerning D-band periodicity. This study highlights the importance of studying collagen D-band periodicity and proves that AFM is a powerful tool for investigating a number of different properties related to collagen D-band periodicity.

## 1. Introduction

### 1.1. General

Collagens comprise about thirty percent (30%) of the whole protein in mammals and they have been recognized as very promising substances for complementing the structure of biomaterials so that they can better interact with biological systems [1,2,3]. Among the vertebrate collagen superfamily, which consist of more than 50 collagens and/or collagen-like proteins [4,5], the greatest interest is presented by collagen type I. This fibril-forming collagen is the most abundant in mammals, including humans, and fulfills unique characteristics, including self-assembly [6].

In order to enhance our understanding concerning collagen’s role in a wide range of functions or diseases and collagen possible applications, an extensive investigation at the nanoscale of the surface properties and structure is demanded. Atomic Force Microscopy (AFM) arises as a novel technique for the nano-characterization of biological samples and biomaterials [7,8]. AFM was invented in 1986 by Binning et al. [9], and the first commercial AFMs started to appear in the market in the beginning of 1990s [10]. Soon after its development, researchers worldwide started using it for nano-imaging and mechanical properties characterization of a wide range of specimens, including biomaterials and biological samples [11,12].

In this mini-review, we first present collagen and more specifically collagen superfamily, collagen type I, collagen-related pathological conditions and collagen-based biomaterials. Then, we introduce a unique collagen nano-characteristic, the collagen D-band periodicity. Recognizing that only few available microscopy techniques can assess the collagen D-band, we highlight the use of AFM technique by introducing its working principle and apparatus and we then focus on its use on the investigation of collagen. Subsequently, we present a systematic search that was performed on databases (Pubmed and Scopus), as well as on other sources (Google, author personal library), to identify relevant research articles that use AFM for studying collagen D-band periodicity.

### 1.2. Collagen

#### 1.2.1. Collagen Superfamily and Collagen-Related Pathological Conditions

The superfamily of vertebrate collagen includes more than fifty collagen and collagen-like proteins [4,5]. All the members of this superfamily are identified by the same structure of the collagen molecule, consisting by a triple-helix from 3 polypeptide chains [5,13]. The characteristic of each polypeptide chain is the repeating pattern of amino acids (Gly-X-Y), where X and Y can be any amino acid. So far, 28 different collagens have been identified in the human body (a group of the collagen superfamily) with different structures and functions. There are different subgroups, such as the fibril-forming collagens, among which the type I collagen is of most interest [13,14]. Other members of the fibril-forming collagens are the collagen types II, III, V, XI, XXIV, and XXVII.

Generally, collagen and collagen mutations are related to many diseases, such as osteogenesis imperfecta, chondrodysplasias, osteoporosis and a number of syndromes (e.g., Ehler–Danlos, Alport, Knobloch). Furthermore, structural variations of collagen at the nanoscale are related to a number of pathological conditions [15,16,17]. In addition, collagen alterations in terms of structure, orientation and mechanical properties have been shown to have a significant role in desmoplastic solid tumors, such as breast and pancreatic cancers [18,19,20,21].

#### 1.2.2. Collagen Type I

Collagen type I is member of the fibril-forming subgroup of the collagen superfamily. Over 90% of the collagen in humans is type I as it is the major protein in the extracellular matrix (ECM). Collagen type I can be found mainly in skin, tendon, vascular ligature, organs and it is the main part of the organic part of bone [22,23]. The molecules of type I collagen form rod-shaped triple helices that are assembled in order to form fibrils [4,24]. The molecules follow a quarter-staggered fashion packing, which leads to the formation of the so call D-band periodicity [1,5,25,26,27,28]. This D-band periodicity is a repeating banding pattern of about 67 nm (depending on the different tissue) and includes gap and overlap regions. Collagen fibrils form bundles and fibers by appropriate alignment [4,24,29]. The fibrils of collagen type I play the role of the elementary building blocks in a wide range of collagen-rich tissues [30,31].

Depending on the tissue, collagen type I fibrils present different morphologies, properties, and have a crucial role in different functions, such as scaffolding and mechanical strength [1,4,32]. For example, in the case of tendons, collagen fibrils have a lateral packing and present a uniform distribution of diameters [33]. On the other hand, in skin, the collagen type I fibrils are randomly oriented and present the form of loosely interwoven and wavy bundles. However, one unique characteristic for collagen type I fibrils is the presence of the so-called D-band periodicity.

#### 1.2.3. Collagen D-Band Periodicity

The packing of collagen type I molecules follow a quarter-staggered fashion that leads to the formation of a unique banding pattern, the D-band periodicity (also known as D-band, D-periodicity, D-spacing) [1,5,25,26]. Generally, the collagen D-band periodicity is considered a unique nanocharacteristic of all fibrils-forming collagens. However, there is a debate whether the length of the D-band periodicity is identical for all fibrillar collagens [34,35]. For instance, some researchers state that there is not a common D-band periodicity among fibrillar collagens [36]. These arguments are based mainly on electron microscopy studies where different D-band patterns were found on different collagen types (collagen type I, II and III), both on positively [37,38,39,40] and negatively [41] stained specimens. In addition, different patterns have been found in studies that compared native collagen type I with type II [42], and reconstructed fibrils type I and V [43]. Furthermore, a D-period of 65 nm has been reported in tissues rich in collagen type III, such as dermis [44] and cornea [45]. It must also be noted that for type I collagen, a fibrillar variant the so-called Fibrous Long Spacing Collagen (FLS) has been reported [46,47,48,49]. These fibrils are characterized by significant larger values in D-band periodicity, typically 200–300 nm. FLS were first reported in in vitro experiments [50], but subsequently, it was found in a number of pathological and normal tissues [51,52,53]. Nevertheless, in this review, we focus on collagen type I D-band periodicity. The D-band periodicity plays a significant role in the collagen type I fibrils mechanical properties and the cell-collagen crosstalk, while it has been associated with a wide range of pathological conditions [27,28,54,55].

The length of the D-band periodicity is tissue-dependent, but in all cases includes gap and overlapping regions [1,5,27,28]. Fibrillar collagens are generally characterized by a D-band periodicity of 64–67 nm depending on the tissue [1]. A number of the variations of the D-band periodicity that have been reported in the literature is presented in relevant articles [56]. For example, some of the values that have been reported using different microscopy techniques are: 64.6 ± 5.3 nm (55–80) for human skin [57], 67.7 ± 0.9 (central zone) and 71.3 ± 0.4 (distal zone) for vitrified predentin [58], and 54–75 nm demineralized dentin [59] (variations in D-band periodicity are also presented in the Results). Furthermore, it has been reported that a D-periodicity of 65 nm it can be found in corneal stroma. One explanation is that the fibrils are more hydrated and, as a result, the molecules are tilted by about 15^o^ to the fibril axis, leading to a reduced axial periodicity [60]. In addition, as mentioned previously, some researchers state that tissues that are rich in collagen type III, such as cornea and dermis, present the 65-nm D-band periodicity [44,45]. In the literature, frequently, the D-band periodicity is introduced as a single value of 67 nm, but as it has been discussed this is not true [56]. Both the collagen type and the tissue play a role. So, it can be said that collagen D-band periodicity is collagen-type and tissue-dependent.

Collagen D-band periodicity has been correlated with fibrils’ mechanical properties, collagen–cell interactions and a number of pathological conditions [27,28,54,55]. It has been shown that cells respond to this periodic pattern and, for example, cell elongation along collagen fibrils/fibers major axis has been correlated with the D-band periodicity orientation [54]. In general, the so called “contact guidance mechanism” has been associated with the motion of the cell along the axis of fibrous features, such as the fibrous proteins (including collagen) of the ECM [61] and the cells’ morphodynamics respond to both surface characteristics and mechanical properties of the surrounding environment [61]. According to the literature, there is a limiting threshold regarding the response of the cells on the contact mechanism, while it has been shown that, at least for fibroblasts, this threshold is ~35 nm [62]. However, the exact mechanism of cell-collagen and/or cell-collagen-based biomaterials is not yet fully clarified.

#### 1.2.4. Collagen-Based Biomaterials

Since collagen possesses unique properties, such as non-toxicity, bio-compatibility, bio-degradability and the ability for self-assembly [6], it has been identified as unique biomaterial for the development of novel biomaterials [1,2,3]. For example, controlling the self-assembly process of collagen can lead to the development of collagen-based biomaterials that can be used as in vitro models of collagen-rich tissues. In addition, it has been demonstrated that collagen-based surfaces with well-organized geometrical features, such as aligned fibers and or porous structures, can guide cell behavior towards a better performance [63,64]. The control of cells movement has attracted significant research interest as it has been associated with specific biological processes, such as wound-healing and metastasis.

Although the process of collagen self-assembly is entropy-driven and the basic principles are known [1], the exact mechanism is not well defined [31] and the development of collagen-based biomaterials with tunable and/or pre-determined characteristics and properties remains a challenging task. The formation of the D-band periodicity is a consequence of the self-assembly process. In vivo, the cells’ (mainly fibroblasts) membrane includes recesses, which appropriately arrange thin collagen fibrils. In the case of the in vitro formation of collagen fibers with D-band periodicity, the mechanism is modified as the self-assembly occurs in the absence of cellular control and a number of growth steps, both linear and lateral, take place [65].

**Figure 1:**
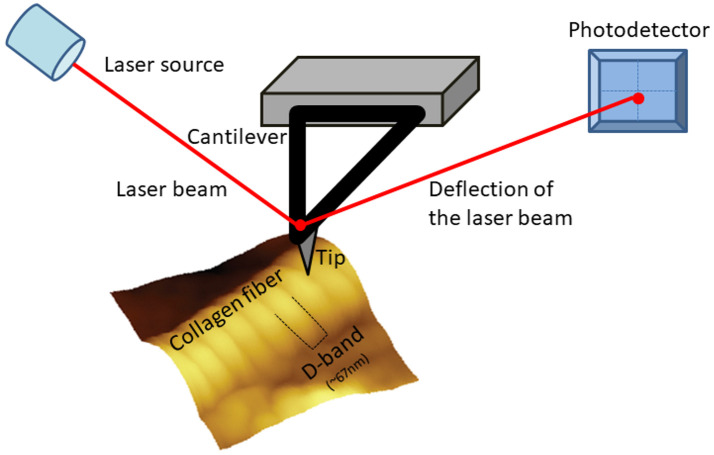
An illustration of an Atomic Force Microscopy scanning a collagen fiber.

As the majority of the biological reactions occur on interfaces and/or surfaces and specific biological processes, including cells’ proliferation and adhesion, are influenced by the nano-characteristics of the biomaterials, the features of the biomaterials’ surface are of pivotal importance in bioengineering and biomedicine [66,67]. Consequently, the nano-characterization of the surface properties of collagen and collagen-based biomaterials is of crucial importance. What is more, the formation of collagen-based biomaterials is not straightforward and that is why novel techniques are required for evaluating and studying the properties of the final material. One of the major tools for these purposes (the characterization of collagen and collagen-based biomaterials), is Atomic Force Microscopy (Figure 1) that we present in the next sections [10,68,69,70]. However, a number of other techniques can be used for characterizing and imaging collagen and collagen-based biomaterials.

#### 1.2.5. Imaging Collagen and Collagen-Based Biomaterials

Collagen and collagen-based samples can be imaged with a variety of imaging techniques, including optical and electron microscopy techniques and Atomic Force Microscopy (AFM). Optical microscopy possesses the advantages of the non-invasiveness and the minimum sample destruction due to light–collagen interactions. It must be noted that there are some specific types of optical microscopy that present selectivity on collagen, such as second harmonic generation (SHG) [71,72,73,74,75] and polarized microscopy, especially when combined with picrosirius red staining [76,77,78]. SHG is a nonlinear optical microscopy technique and no staining is demanded, while for picrosirius red staining polarization microscopy, fixation and staining is demanded. However, for all the light microscopy techniques, it must be taken into account that the size of the collagen fibrils is smaller or at the limit of optical microscopy. Of course, the D-band periodicity is much smaller of the resolution of optical microscopy. Furthermore, in tissues, fibrils are assembled into bundles and sheets and the imaging of individual fibrils is impossible with a light microscope. However, in cases that high resolution is demanded, optical microscopy techniques are used. In addition, in many cases, correlative microscopy or different microscopy techniques are combined, such as AFM and SHG [25,26]. Concerning, electron microscopy techniques, transmission electron microscopy (TEM) and scanning electron microscopy (SEM) were for many years the major imaging techniques of collagen and collagen-based biomaterials, especially TEM that presents higher resolution [79,80,81]. Electron microscopy techniques enable also the measurement of the fibrils’ length, the fibril volume fraction (FVF) and the 3D organization of the fibrils [79]. The D-band periodicity was originally observed in the early 1960s on electron micrographs that led the introduction of the D-band periodicity (D-stagger), and subsequently, the model that describe it with the gap and overlap regions [82]. Studies with TEM showed that the average D-band periodicity is in the range of 64–70 nm [83]. Unfortunately, electron microscopy techniques present some significant limitations. First of all, during sample preparation biomolecules such as collagen irreversibly denature as a consequence of the harsh chemical fixation and the staining procedure [84]. In addition, the specimen–electron beam interaction may destroy collagen ultrastructure. Some of these drawbacks may be overcome with cryo-electron microscopy techniques that protect biomolecules’ native ultrastructure and have the advantages of electron microscopy [85]. However, the fact that these techniques require a highly specialized equipment, complicated analysis strategies, complicated preparation protocols (e.g., freezing tissues) and are characterized by poor signal-noise ratio that minimize their applications. Finally, AFM is a high-resolution surface technique that can combine both imaging and mechanical properties characterization of biological samples. Minimum sample preparation is demanded and it can be performed under different environmental conditions. As the focuses of this review is the characterization of collagen D-band periodicity with AFM, detailed information about AFM and its applications concerning collagen are presented in the next sections.

### 1.3. Atomic Force Microscopy 

#### 1.3.1. General

Atomic Force Microscopy (AFM) was developed in 1980s [9] and is member of the scanning probe microscopies family. AFM measures the probe (a tip mounted on a cantilever) surface interactions in order to assess information concerning the topography and the mechanical properties of the sample at the nanoscale. Since its invention, AFM has arisen as a fundamental nano- and microscopy technique, as it possesses a number of advantages compared to other microscopes (such as optical microscopy, Scanning and Transmission Electron Microscopy). AFM can operate in different environmental conditions, with different modes, that can offer a wide range of information from surface topography to mechanical characterization of specimens that require minimum preparation [10,86,87]. These features make it very attractive for biological samples, including collagen- based tissues and biomaterials.

#### 1.3.2. AFM Basic Principles

AFM is very different than conventional microscopes as it has no lenses of any kind. Actually, AFM “feels” rather than “looks” at the sample surface [10]. An AFM system uses a probe in order to collect the specimen information. The AFM probe is a tip (with different shapes depending on the application) mounted on a cantilever, which are attached on a chip. Piezoelectric elements are used for the accurate scanning of the tip over the sample. The movement of the tip is being monitored by using a laser beam. A laser source emits a laser beam in the backside of the cantilever. The deflection beam is then detected by a photodiode (a photodetector). During the scanning procedure the cantilever bends, due to the tip-sample forces, and the photodetector monitors the movement of the tip by the changes of the laser spot position. The photodetector, which is a position-sensitive detector, translates the laser deflection into an electric signal that is used for the formation of a 3-D image of the surface [9] or it is used for force spectroscopy purposes depending on the used mode.

#### 1.3.3. AFM Modes

One of the more significant characteristics of AFM is that it can operate in a wide range of different modes, and as a result, it can offer both qualitative and quantitative information concerning bio-samples [70,87,88], such as nano-topography and mechanical properties characterization [7,89,90,91]. The most widely used AFM modes are the contact mode, the tapping mode, and the non-contact mode (where the tip does not come in contact with the sample surface). In contact mode, the tip is always in contact with the sample surface and topographical maps are provided. In addition, during contact mode, Lateral Force Microscopy (LFM) imaging can be performed. LFM offers information relevant to the surface friction which is a consequence of the material’s surface inhomogeneities. In tapping mode (also known as intermittent mode, dynamic contact mode and AC mode), the cantilever oscillates at its resonance frequency. Tapping mode can be used for acquiring topographical images, while it can simultaneously capture phase images [10]. Phase imaging use as contrast the phase lag between the cantilever’s driving signal and its output signal. Phase images are useful in order to study variations in composition, friction, adhesion and viscoelastic properties of heterogeneous specimens [59,69]. The information from phase imaging goes beyond topographical features [59,69]. Tapping mode is preferred in the case of the biological specimens as they are quite soft, and this mode is less destructive as lateral and frictional forces are minimized [7,11]. Finally, AFM can also operate in non-contact mode. This mode is not frequently used in biological samples as it is quite difficult to perform. During this mode, the AFM tip does not come in contact with the sample surface and uses only attractive forces.

AFM can also be used for nanomechanical properties characterization of the samples with force spectroscopy. In this experimental procedure, the AFM probe acts as a force sensor [92] for assessing the mechanical properties of the sample with different modes [93]. For instance, the AFM nanoindentation procedure (where indentation–force curves are formed) [94,95,96] or the force scanning mode [97] can be used for acquiring information regarding the samples stiffness (in terms of Young’s modulus values or stiffness maps) [69,89,91,98,99]. For acquiring the specific values, mechanical models are used, such as the Hertz model, which is the most widely used, while commercial or home-built software are used for the fitting the mathematical expressions [100,101].

#### 1.3.4. AFM Limitations

It must be noted that AFM, as the majority of the microscopes, is also characterized by a number of drawbacks [102]. First of all, AFM is a surface imaging and mechanical properties characterization technique. Although AFM can provide information concerning the *z* axis (is one of the few microscopes that provide 3D maps of the surface), this ability is limited to the length of the tip. So, it provides only characterization of the surface and not the whole specimen in the *z* axis. In the case of the collagen-based materials, AFM cannot measure the organization of the fibrils in the three dimensions or the fibril volume fraction (FMV) [79]. Furthermore, in the cases of the biological derived collagen-based specimens (such as tissue sections), the characterization of the surface is very challenging as the specimen collection procedure or preparation methodology (e.g., needle biopsy collection, section with surgical blades) may destroy the collagen ultrastructure. However, the combination with cryosectioning techniques enables the visualization of the ultrastructure of unstained specimens [103], while more complicated specimen preparation techniques have been proposed in the literature [104,105]. One more limitation is that AFM systems can image a single nano-sized image at a time. The size of the maximum size of the image depends on the different AFM scanners, which are different depending on the manufacturer. As a result, information, such as the collagen fibril length, cannot be assessed, but it does not affect features such as the D-band periodicity. Furthermore, AFM imaging is characterized by low scanning time, which can cause thermal drift on the sample. This limitation is overcome by novel developments in AFM modes and techniques, such as the fast/high-speed scanning [106]. One more limitation, concerning collagen characterization is related to the sample preparation and the need for the specimen to be firmly attached to a substrate. As the AFM tip exerts forces on the specimens, loosely attached samples cannot be characterized. In the case of tissue specimens, biocompatible glues are used, but careful handling is demanded so as to protect the surface. On the other hand, for in vitro self-assembled collagen fibrils, physical adsorption of the collagen solutions on a flat substrate, such as mica, is usually used. Another source of drawback arises when collagen characterization is performed in air as a layer of water condensation and other contamination often covers both probe and sample. This leads to attractive forces that affect imaging. In order to overcome this limitation, AFM characterization under liquid conditions (e.g., saline buffers) is usually used. Finally, a source of possible limitation arises from the size and shape of the AFM tip. Inappropriate, damaged or contaminated tips may influence topographical characteristics, such as the D-band periodicity and the relevant measurements. Currently, a number of manufactures offers a wide range of AFM tips, with different shapes, sizes and for different environmental conditions and modes, so as the appropriate ones to be selected. In addition, calibration gratings can be used for assessing tips’ performance. Overall, as the AFM technology matures, new advances and developments help researchers to overcome the majority of the limitations of this technique.

#### 1.3.5. AFM and Collagen

One of the major advantages of AFM in biology and bioengineering studies is the fact that it does not demand significant sample preparation. For example, for the AFM characterization, it is not necessary to coat or label the specimen with dyes/antibodies, while depending on the sample, dehydration is not mandatory [11,12,21]. Furthermore, AFM can operate both in air and liquid [86], while also experiments under vacuum conditions have been performed. Concerning the application of AFM on collagen-based samples both imaging and mechanical properties characterization have been applied in a wide range of samples, from pure collagen to collagen rich-tissues and biomaterials. AFM scanning does not affect or destroy the collagen structure, while AFM resolution can provide information from molecules to individual fibrils/fibers [6,107]. AFM has been applied for investigating different properties of collagen, including collagen structure, the role of collagen in a number of pathological conditions and collagen–cell interactions. In the next section, we present some of the more representative studies concerning the focus of this paper which is the use of AFM for health purposes.

## 2. Materials and Methods

For this systematic review, articles were searched through the PubMed and Scopus databases, while secondary articles that were obtained from the articles, which were originally found, were also used. For the searching procedure in the databases, chronological limits were not used and the used keywords are presented in Table 1.

The articles which have been chosen to be used in this systematic review, are based on the following criteria:

Inclusion-exclusion criteria

(1) No systematic studies or review publications;
(2) No conference proceedings;
(3) Be in English language (publications in any other language were not included);
(4) Studies that did not use AFM were not included;
(5) Studies that did not study the D-band were not included.

## 3. Results

**Figure 2:**
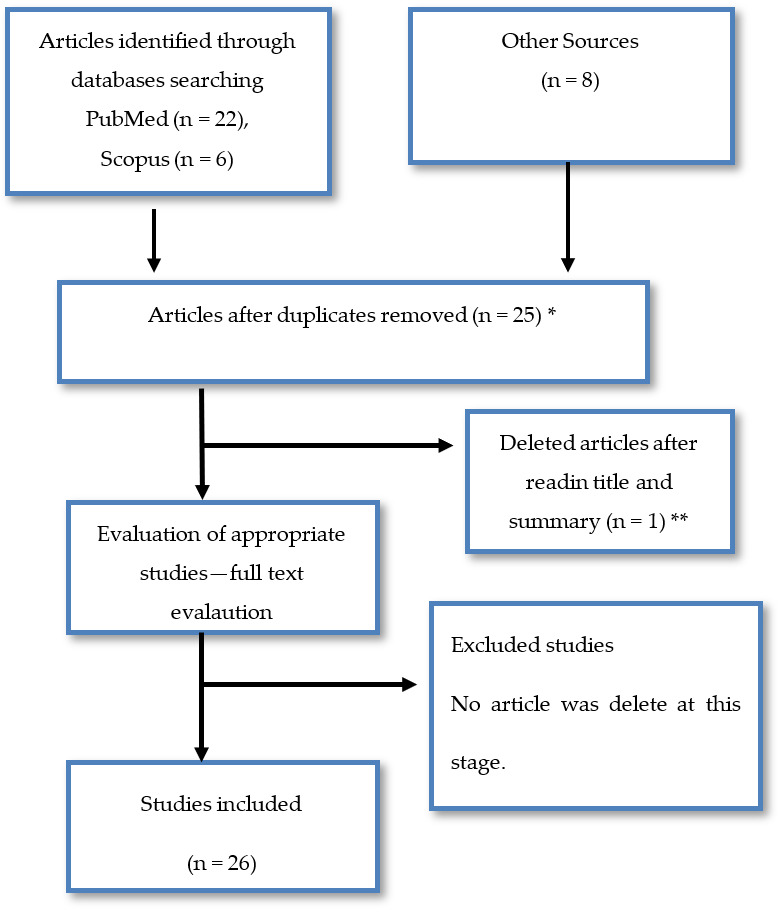
Flow diagram. Schematic representation of the searching procedure and the evaluation of the articles based on the inclusion and exclusion criteria. (* After duplicate removal, 2 papers were only found on Scopus, 4 articles were only from other sources and the rest only from Pubmed. ** The Hiyama et al. (1998) [81] paper was removed as a paper that does not study collagen, but is focused on type A fibrils of the mouse tectorial membrane.)

The studies that were found were evaluated on the basis of the admission criteria at each stage as well as the final number of studies determined on the basis of their suitability for the use of the bibliographic review were presented on a flow diagram (Figure 2). As it can been seen from the flow diagram, from the 36 articles that were originally found, only 26 satisfied the inclusion criteria.

The 26 articles were studied and Table 2 presents the major information that was obtained from the 26 articles that met the inclusion criteria. The articles in the table are presented in chronological order. The major information that was collected included:

- Year of publication (and authors);
- Type of collagen that the researchers used in their work;
- Type of AFM mode that was applied;
- Environmental conditions under which the experiments were conducted;
- Major results, mainly concerning D-band periodicity.

## 4. Discussion

**Figure 3:**
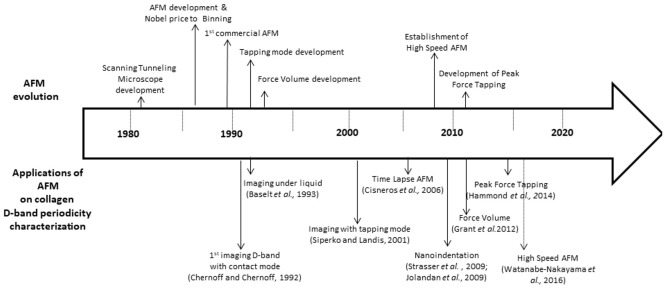
Time-line of AFM development and relevant techniques used for studying collagen D-band periodicity.

From Table 2, we can see that a number of different modes of AFM have been used for studying the collagen D-band periodicity, and AFM technological developments were used shortly after their invention (Figure 3). Only a few years after the invention of AFM, it was used for studying the collagen D-band periodicity. AFM was introduced to scientific community by Binning, Quate and Gerber in 1986 [9] (with the first commercial microscope to be introduced in 1989) and in 1992 Chernoff and Chernoff used AFM for studying D-band periodicity in collagen fibrils from type I bovine skin collagen. The researchers do not mention what mode they were using, as at that time, only the contact mode was developed. The tapping mode was first introduced in 1993 [135], and consequently, all the publications until that time were probably using the contact mode. In the papers that were found, the first direct mention of the use of tapping mode for studying collagen D-band periodicity was performed by Siperko and Landis in 2001 [113]. Furthermore, the majority of the studies in the first years after the development of AFM were conducting experiments in air.

Generally, AFM under liquids/buffers is a very widely used technique, but even in our days, in the cases of biological samples, it is a challenging procedure. Very early, in 1993, Baselt et al. [109] mentioned the use of AFM both under air and water for imaging the D-band periodicity in a native rat tail. The next use of AFM under a buffer was reported by Cisneros et al. in 2006 [116], where the in vitro establishment of fibrils with D-band periodicity was reported. Actually, the authors in this publication used the time-lapse technique, where images of the same area were obtained within specific time intervals. Concerning technological developments, in 2014, Hammond et al. reported the use of peak-force tapping mode (this mode is exclusively developed and used by one of the major AFM-system manufacturers—Bruker) for studying the collagen D-band periodicity from tibiae and tails from control and diabetic rats. Subsequently, concerning the use of different techniques for investigating collagen D-band periodicity, Spitzner et al. reported the use of MUSIC-mode (multi-set point intermittent contact mode, which is no so widely used) and the nano-indentation in order to investigate the impact of water content on reconstituted collagen type I fibrils, specifically their shape and mechanical properties [127]. Then, there is a number of studies that use not only imaging techniques but also force–distance curve measurements in order to acquire nanomechanical properties [91,118,119,127,130,131], including the force volume imaging (that also collects mechanical properties) [28]. Finally, in 2016, Watanabe-Nakayama et al. [129] conducted experiments applying the high speed AFM for investigating how clostridial collagenase moves along collagen fibrils, while Gisbert et al. very recently (2021) developed a high-speed bimodal AFM that was used to study the initial stages of collagen self-assembly [106]. For this short discussion in this paragraph, it is clear that AFM technological developments historically were very early used in order to study collagen and collagen D-band periodicity.

The papers that were found showed that a number of different collagen sources were used, including collagen from:

- Mouse (tails [128,134], bones [27]);
- Rat (tail tendons [28,110,111,112,114,125,129,131]);
- Tibiae [125];
- Turkey (tendons [113]);
- Ovine/sheep (dermal [121,122,123]);
- Pig (femur [128]);
- Cow/ox/steer (bovine skin/dermal/hide [108,109,116,118,124,127,130], Achilles tendon [91,119,126], tendon [132], tail [133], also monomeric collagen without any more specifications of origin [106]);
- Human (heart valves [115], fibrous joints [120], skin [122], tendons [122]),
- and even collagen from sponge [117].

The specimens from these sources were used either direct as biopsies from the species or as reconstructed collagen from solutions or lyophilized collagens (lab-developed or commercial products). Consequently, this highlights the presence of the D-band periodicity on fibers/fibrils of collagen from different species and the ability of AFM to study collagen nanocharacteristics and especially collagen D-band periodicity in a wide range of specimens. It is interesting that appropriate techniques enable the in vitro development of collagen fibrils/fibers with naturally occurring characteristics such as the D-band periodicity.

More interestingly, a number of studies investigate the D-band periodicity in terms of the relation of relevant pathological conditions or biological process. Specifically, Aragno et al. studied the D-band periodicity in correlation to aging [110]. Their results demonstrated that although there was no difference in the band interval, collagen extracted from old rats present lower widths and heights and the depth of gap between two overlaps showed a higher mean value. Odetti et al. conducted experiments using diabetes murine models [112]. The D-band periodicity in the fibrils from the tails did not present significant differences, but alterations were identified in the gap depth, highlighting that axial organization is not modified by non-enzymatic glycation. Concerning diabetes, Wang et al. investigated nerve collagen and the results, one more time, demonstrated that no alteration occurred in D-band periodicity, although fibril diameters were becoming larger in the case of diabetes. In addition, the differences in collagen fibers from bones and tails in diabetic rats was performed by Hammond et al. [125]. The values of the D-band periodicity in collagen fibers from bones and tendon presented higher values in diabetic rats than control. On a different direction, Wallace et al. tried to study D-band with AFM in correlation to osteogenesis imperfecta. Again, the average spacing remained unchanged, although it presented greater variability compared to the control group [27]. On the other hand, in two research papers, the researchers tried to investigate possible correlation between collagen fibrils characteristics and estrogen depletion [121,123]. Interestingly, among other results, the researchers found that a new subpopulation of fibrils with altered D-spacing was occurring after estrogen depletion [121]. Their results showed that is not the mean value of the D-band periodicity that matters but the distribution of the values that have statistical significance in the case of estrogen depletion which has been correlated with early stage of osteogenesis imperfecta and osteoporosis. Furthermore, Peacock and Kreplak investigated collagen from fibrils under tension and found an increase in D-band periodicity length [132], while Baldwin et al. showed the selective enzymatic removal of material in the non-D-banded regions by matrix metallo-proteinases nine (MMP-9) mainly on damaged fibrils by mechanical overload [133]. Finally, Cauble et al., by using murine models with degenerative discs, found that D-band periodicity distribution was shifted to higher values in the degenerative discs. The rest of the studies focus on the use of AFM on investigating the structure (including the D-band) of collagen fibers/fibrils from different origins, the impact of different parameters, such as the UV irradiation, and the mechanical characterization of the fibrils.

Although the search of relevant literature was performed with a systematic methodology on two of the more widely used and recognized scientific databases (PubMed and Scopus), a number of articles (8) were found through other sources. In addition, for searching the databases, many keywords were used for searching the same term (D-band periodicity). The MeSH does not include any relevant term, and generally, it seems that many different terms are used in the literature and academia. This generates confusion and probably a number of other articles may be already available in the literature but cannot be identified if different keywords are used. The use of a common term for D-band periodicity would be very helpful for identifying relevant research articles. In addition, a personal opinion of the author is that many research articles study D-band periodicity with AFM but relevant keywords are not provided in the title, abstract or keywords of the articles. As a result, these articles are even more difficult to be identified.

## 5. Conclusions

The collagen superfamily includes members among which the fibril-forming collagen type I is the most abundant. Fibril-forming collagens present unique characteristics, among others, the so-called D-band periodicity. This review focused on collagen type I and highlighted that AFM is a unique tool and among the few that can be used for studying collagen D-band periodicity. The literature so far has shown that AFM can be used for obtaining a number of novel information regarding collagen fibers/fibrils structure, the nature and role of D-band periodicity and its correlation with many pathological conditions or biological processes. The new technological developments that are emerging in the field of AFM will further advance the research in this field.

## Figures and Tables

**Table 1:** Search Strategy. This table presents the keywords that were used in order to identify the relevant publications.

Term	Key Words ^1^
Collagen D-band	1. “Collagen D-band” 2. “Collagen D band” 3. “Collagen D-periodicity” 4. “Collagen D periodicity” 5. “Collagen D-spacing” 6. “Collagen D spacing”
7. #1 OR #2 OR #3 OR #4 OR #5 OR #6	
Atomic Force Microscopy	8. “Atomic Force Microscopy” 9. “AFM”
10. #8 OR #9	
11. #7 AND #10	

^1^ There are no relevant MeSH terms. Only “collagen” is an existing MeSH term.

**Table 2:** Major information from the publications that were found through literature search.

A/A	Authors (Year) Reference	Collagen	AFM	Environment	Results Concerning D-Band	
					D-Band Values (nm)	General Results
1	Chernoff and Chernoff (1992) [108]	Fibril-forming and monomeric type I collagen from type I bovine skin collagen	Not specified [contact mode]	Air	70	Collagen fibrils that were imaged with AFM presented the characteristic D-band periodicity (~70 nm). [Probably the first demonstration of collagen D-band periodicity with AFM]
2	Baselt et al. (1993) [109]	Native rat tail and reconstituted bovine dermal type I collagen	Not specified [contact mode]	Air/Water	60–70	The authors performed AFM imaging of the D periodicity (60–70 nm) in collagen type I from native rat tail and reconstituted bovine dermal fibrils. They also found a “minor” period and “microfibrils” that were arranged either parallel to or inclined −5° to the fibril axis.
3	Aragno et al. (1995) [110]	Collagen (type I) from rat tail tendon fibers	Contact mode in height mode	Air	67.0 ± 0.1	Study collagen fibrils at different fibrillogenesis times. Although, the fibrils presented an increasing size with time, the band interval did not change. In addition, the depth of the D-band periodicity remained stable after an initial increase.
4	Odetti et al. (1995) [111]	Collagen (type I) from young and old rat tail tendons.	Contact mode in height mode	Air	Young rats 67.0 ± 0.1 Old rats 66.5 ± 0.1	Collagen from young and old rats presented the same D-band interval, but collagen in the aged rats was characterized by lower widths and heights. Furthermore, collagen from old rats presented a higher value in the depth of gap between two overlaps.
5	Odetti et al. (2000) [112]	Tendon fibrils (collagen type I) from tails of rats which developed diabetes and from diabetes-resistant rats of the same strain.	Not specified [probably contact mode]	Air at room temperature and a humidity range of 40 ± 50%.	Non-diabetic 67.0 ± 0.1 Diabetic 67.0 ± 0.1 Glucose-incubated fibrils 67.3 ± 0.2	They studied modification in collagen structure caused by high glycose concentration. Non-diabetic fibrils present differences in radius and gap depth compared to the diabetic and glucose incubated fibrils. Although alterations were observed in gap depth, the D-band periodicity was the same in the studied groups, demonstrating that non-enzymatic glycation did not alter the axial organization.
6	Siperko and Landis (2001) [113]	Collagen and minerals in tendons from turkey (not mentioned what collagen, but in tendons is type I)	Tapping Mode	Air (ambient laboratory conditions)	62 ± 8	AFM images showed numerous mineralized collagen fibrils that were align parallel to each other and they were presenting the characteristic D-band periodicity. In addition, in some occasions, mineral plates were observed between and in intimate contact with collagen fibrils.
7	Wang et al. (2003) [114]	Nerve and tail tendon collagen from control and diabetic rats. (Sprague-Dawley rats—SD and biobreeding rats—BB.) Collagen type is not specified (probably type I)	Not specified [probably contact mode]	Air	63–69 Sciatic nerve SD rats Control 66.8 ± 3.8 (epi) 63.6 ± 3.6 (endo) Diabetic 64.7 ± 4.3 (epi) 63.8 ± 4.9 (endo) BB rats Control 67.8 ± 3.2 (epi) 65.5 ± 4.0 (endo) Diabetic 68.1 ± 2.8 (epi) 68.5 ± 3.8 (endo) Tail tendon BB rat Control 69.4 ± 2.4 Diabetic 67.9 ± 2.2	Collagen fibrils from diabetic rats presented higher diameters compared to collagen from control rats. No significant changes were observed in D-band periodicity that varied from 63 to 69 nm.
8	Jastrzebska et al. (2006) [115]	Collagen structure in human aortic, mitral, tricuspid and pulmonary heart valves (the collagen component of the specimens is predominantly type I (74%) and type III (24%))	Contact mode	Air	70–80	The valves with different origins demonstrated a significant heterogeneity as far the collagen fibrils’ surface topography. Almost all the fibrils acquired the characteristic D-band periodicity, with the band interval presenting a wide range of values.
9	Cisneros et al. (2006) [116]	Solubilized bovine dermal collagen (type I)	Time-lapse Tapping Mode	Buffer	~67	In this study, the researchers achieved the in vitro assembly of collagen fibrils with the natural occurring D-band periodicity of ~67 nm. It was shown that the assembly is a two-step process, where the molecules firstly assemble with each other and, subsequently, the molecules are rearranged into microfibrils.
10	Heinemann et al. (2007) [117]	Ιsolated fibrils of Chondrosia reniformis sponge collagen.	Tapping mode	Air	Chondrosia collagen 67–69 (but consisting by different gap and overlap zones)	In this investigation, by using AFM, the morphology of collagen from sponge was compared with the morphology of other fibril-forming collagens. It was found that sponge collagen presented a quite different D-band periodicity consisting of overlap zones followed by 2 identical gap zones.
11	Strasser et al. 2009 [118]	Collagen solution from calf skin (collagen type I, in vitro fibrous long spacing collagen fibers).	Non-Contact mode Nanoindentation	Air (with control humidity)	78	In this work, the authors report the AFM microdissection technique, where they cut collagen fibrils in order to study both the core and the outer shell of the fibril. The results concerning collagen fibrils’ structure demonstrated that the D-band periodicity can be found also in the core and not only on the shell of the fibrils. In addition, nanomechanical measurements indicated that the D-band periodicity both in the core and the shell have the same Young’s modulus values.
12	Jolandan et al. 2009 [119]	Type I collagen fibrils from bovine Achilles tendon	Tapping mode Contact mode Dynamic Nanoindentation Static Nanoindentation	Air	~67 nm	The authors demonstrated the mechanical heterogeneity along the axial direction of a single isolated collagen fibril from tendon and showed that, within the D period, the gap and overlapping regions have significantly different elastic and energy dissipation properties, correlating the significantly different molecular structures in these two regions
13	Hurng et al. (2011) [120]	Collagen from human fibrous joints (specific type of collagen is not provided).	Contact mode	Air and hydrated conditions	Not provided	AFM demonstrated structural reorganization of the periodontal ligament (PDL), collagen spacing, organic-dominant areas at the PDL- cementum and PDL-bone entheses and within cementum and bone.
14	Wallace et al. 2011 [27]	Collagen type I from murine femurs in strains that present D-band periodicity alterations related to osteogenesis imperfecta	Tapping mode	Air	Wild Type 67.6 Heterozygous (Brtl/+) mice 67.4	AFM was used to image and quantitatively characterize the D-band periodicity of type I collagen fibrils related to osteogenesis imperfecta.
15	Fang et al. (2012) [121]	Ovine dermal sections (type I collagen)	Contact mode	Air	Ovariectomized61.9 Control 62.3	Using AFM, it was shown that after estrogen depletion, nano-scale morphological alterations of dermis collagen fibrils occurred. After 2 years of ovariectomy in ovine dermal samples, a new subpopulation of fibrils with D-band periodicity was found. In addition, it was found that the overall width of the distribution was increased.
16	Grant et al. 2012 [28]	Collagen type I fibrils from rat tail tendons	Force Volume Imaging	Air	67.4 ± 1.8	The authors conducted low-frequency dynamic mechanical analysis on individual collagen type I fibrils in order for elastic and viscous responses to be correlated to the D-band periodicity.
17	Fang et al. (2012) [122]	Specimen from ovine dermis and bone. Human skin biopsies and tendons were also acquired. (Collagen type I)	Contact mode	Air	58–69	In this research work, it was found that the D-band periodicity of collagen fibrils, within a single bundle, from specimens of different origins, was nearly identical and frequently differs by less than 1 nm. In addition, similarity in D-band periodicity for up to 40 μm in bundle length and width was observed, and it was demonstrated that D-band periodicity presents differences at the bundle level, independent of species or tissue types (dermis, tendon, and bone).
18	Erickson et al. (2013) [123]	Type I collagen morphology in disease models from dermal sheep skin of estrogen depletion and osteogenesis imperfecta	Tapping mode	Air and water	Air 63.1 ± 1.9 Water 62.2 ± 2.0	In this work, a quantitative approach that combined AFM and 2D Fast Fourier Transform was used for measuring D-band periodicity. The authors demonstrated that in the case of estrogen depletion (that is correlated with osteogenesis imperfecta and early stage of osteoporosis), it is the D-band periodicity that presented statistically significant differences and not the D-spacing mean.
19	Gudzenko and Franz (2013) [124]	Bovine collagen type I monomers Mixed with ITC- conjugated bovine collagen type I	Contact mode	Liquid (PBS)	67	The authors of this study investigated the adherent cells/ECM interactions at the basal cell side. A number of different substrates were used, including collagen substrates presenting natural characteristics, such as the D-band periodicity.
20	Hammond et al. (2014) [125]	Right tibiae and tails from control and diabetic rats (collagen type I).	(Peak force) tapping mode	Air	Bone Control 66.1 ± 0.8 Diabetic 66.5 ± 1.5 Tendon Control 68.4 ± 0.2 Diabetic 68.6 ± 1.2	Diabetic bones had had a noticeable different distribution of collagen D-band periodicity than controls. The distribution from the diabetic bones was characterized by variability and higher values. The shift to higher values in D-band periodicity distribution was more evident in the case of tendons. Diabetes in rat promotes alterations to the nanoscale morphology of collagen, which results in nano-mechanical and compositional effects in bones.
21	Stylianou et al. (2014) [126]	Collagen solution and thin films formed with spin coating procedure from type I collagen from bovine Achilles tendon.	Contact and Tapping Mode	Air	67	UV irradiation was applied for both collagen solution and thin films. For short irradiation times, AFM imaging showed modification in surface roughness. Additionally, different effects were found when UV irradiation was performed on collagen solution compared to irradiation on thin films. Fibroblasts responded on surface alterations after UV irradiation of both collagen solution and films. Long irradiation intervals deformed fibrils revealing a number of inner shells. In addition, it was found that collagen D-band periodicity is presented not only in the outer shells but also to the inner ones.
22	Spitzner et al. (2015) [127]	Type I collagen isolated from bovine hide	MUSIC-mode AFM/ Nano-indentation	Air with control humidity	67	In this study, they investigated the impact of water on collagen type I. It was found that during swelling, gap and overlap present a difference in the water uptake. This result is direct evidence for different amounts of bound and free water within the gap and overlap regions. On the one hand, in the dry state, the D-band periodicity that is recorded by AFM imaging is due to height corrugations along a fibril’s axis. On the other hand, in the hydrated state, the surface of the fibril is smoother and D-band periodicity presents different nanomechanical properties in the gap/overlap regions.
23	Wallace (2015) [128]	Specimens from the anterior diaphysis of a pig femur. Samples were demineralized to expose collagen. Also, mouse tail tendons were characterized. (collagen type I)	(Peak force) tapping mode	Air (dry and wet samples)	Control 64.9 ± 0.4 Fixed 65.8 ± 0.2	The aim of this work was to explore whether the fixation of bone maintain collagen ultrastructure. Specimens were studied with AFM and the results showed that after fixation D-band periodicity variability was decreased. In addition, it was found that D-band periodicity had higher average periodicity compared to control samples. Furthermore, data from tendons showed that after fixation of drying do not significantly affect collagen structure as it presents characteristics similar with those of its native state.
24	Kontomaris et al. (2015) [91]	UV irradiated type I collagen from bovine Achilles tendon	Force–distance (FD) and contact mode AFM imaging	Air	67	The results showed that the UV irradiation influence the height level differences between the gap and overlap regions. In addition, it was found that the Young’s modulus values were reduced after irradiation, confirming that UV affects collagen fibrils mechanical properties.
25	Watanabe-Nakayama et al. (2016) [129]	Rat tail type I collagen	High speed Tapping mode	Water	67 Overlap 36.18 Gap 30.82	They studied the movement of clostridial collagenase along collagen fibrils
26	Uhlig and Magerle (2017) [130]	Reconstituted type I collagen fibrils (acid-extracted, purified type I collagen from bovine calf hide)	force–distance (FD) and amplitude-phasedistance (APD) measurements	Air with controlled relative humidity	[not measured].	The research results of this study showed that differences in the mechanical properties of the gap and ovelrap regions are only observed in the top 2 nm but not in the fibril’s bulk.
27	Stylianou et al. (2018) [131]	3D collagen type I gels (rat tail)	force–distance (FD), tapping and contact mode AFM imaging	Ari histological sections from collagen gels	[not mentioned]	Developed protocol for imaging and measuring mechanical properties of collagen fibers with D-band with AFM.
28	Peacock and Kreplak (2019) [132]	Tendon from the forelimb of an 18–24 months old steer. [Collagen type is not mentioned but tendons are rich in collagen type I].	Force–distance curves (obtained in Peak Force QNM)	Liquid (PBS)	67 ± 2	In this work, single collagen fibrils under tension were studied. The results demonstrated that upon 5–30% stretching a radial stiffening is observed consistent with the fibrils being under tension. This is correlated with an increase in D-band length. Furthermore, the indentation modulus contrast which is relevant with the D-band periodicity was increased linearly with D-band strain.
29	Baldwin et al. (2019) [133]	Tendons from bovine tails (steers aged 18–24 months). [Collagen type is not specified but tendons are rich in collagen type I].	Tapping mode	Air (hydrated)	[Not measured].	In this work, AFM was used in order to study trypsin and MMP-9 enzymatic removal of material from fibrils which were: (i) untreated, (ii) partially heat denatured, (iii) or displaying discrete plasticity damaged after repeated mechanical overload. The results demonstrated that both enzymes removed material from the two groups and not the untreated groups. Interestingly, the researchers showed that MMP-9 presented selective removal of non-D-banded material, especially in the case of the damaged fibrils.
30	Cauble et al. (2020) [134]	Mouse tails from a murine model of degeneration of the intervertebral disc. The intervertebral disc (IVD) consists of: -the annulus fibrosus (AF), comprised of type I collagen -nucleus pulposus (NP), comprised of proteoglycan and type II collagen.	Contact mode	Air	Nondegenerate annulus fibrosus 61.1 ± 2.9 Degenerate annulus fibrosus 62.6 ± 2.3 Degenerate nucleus pulposus 62.6 ± 2.3	The researchers showed that in the case of degenerative discs, the fibril D-band periodicity distribution shifted to higher values in the annulus fibrosus, as well as in nucleus pulposus. In addition, a novel microstructural feature, collagen toroids, defined by a topographical pit enclosed by fibril forming matrix was observed in the nucleus pulposus. After degeneration alterations were observed, including increase in the number of these structures, while they were reshaping to oval microstructures instead of circular ones.
31	Gisbert et al. 2021 [106]	Monomeric bovine collagen type I	High-speed bimodal AFM	Buffer	67	The authors reported the development of a high-speed bimodal AFM. This system can offer maps with high spatial resolution of the: elastic modulus, loss tangent, topography. The developed microscope was used for investigating the initial stages of collagen self-assembly. The researchers, based on alteration in the physical properties, found 4 distinct stages: (i) nucleation and growth of collagen precursors, (ii) formation of tropocollagen molecules, (iii) assembly of tropocollagens into microfibrils, and (iv) alignment of microfibrils to generate microribbons).

Comments of the author are presented in brackets […]. Abbreviations: AFM—Atomic Force Microscopy, MMP—Matrix metallo-proteinases, PBS—Phosphate buffered saline.

## Data Availability

Not applicable.
